# Effects of fludioxonil, propolis and black seed oil application on the postharvest quality of “Wonderful” pomegranate

**DOI:** 10.1371/journal.pone.0198411

**Published:** 2018-05-31

**Authors:** İbrahim Kahramanoğlu, Mehmet Aktaş, Şerife Gündüz

**Affiliations:** 1 Department of Horticultural Production and Marketing, Faculty of Agricultural Sciences and Technologies, European University of Lefke, Lefke, Northern Cyprus via Mersin, Turkey; 2 Institute of Education, Near East University, Lefkosa, Northern Cyprus via Mersin, Turkey; University of Calcutta, INDIA

## Abstract

Pomegranate fruit consumption has increased rapidly throughout the world, mainly because of its medical and nutritive attributes. Thus, considerable commercial and scientific interest exists in prolonging its postharvest life with non-chemical applications as much as possible to meet the year-round demand for this fruit. The present work aimed to study the effects of black seed oil (0.1% and 0.5%), propolis (0.01% and 0.1%) and fludioxonil (0.06%), with and without modified atmosphere packaging (MAP), on the postharvest quality of pomegranate cv. Wonderful. Treated fruits were stored at 6.5±1 °C and 90–95% relative humidity for 150 days. The results indicated that both black seed oil and propolis treatments significantly influenced the maintenance of fruit weight and quality. At 150 days after storage, the fruit weight loss of the samples treated with MAP + 0.5% black seed oil, MAP + 0.1% propolis and MAP alone were found to be 5.5%, 6.3%, and 9.1%, respectively, whereas the weight loss of the untreated control fruits was 19.8%. Application of either 0.5% black seed oil or 0.1% propolis, especially when combined with MAP, was also effective in controlling gray mold development and slowing the occurrence of chilling injury.

## Introduction

Pomegranate (*Punica granatum* L.) is a traditional crop with a long history of cultivation. However, consumer preference for this fruit was low for many centuries, due to the difficulty of extracting the arils. Since the beginning of the 21^st^ century, many scientific studies have shown the health benefits of pomegranates [1]. The findings of these studies and human dietary changes have caused a considerable increase in the consumption of pomegranate fruit. The harvesting periods of pomegranate fruit generally start in March and August in the southern and northern hemispheres, respectively. Because these periods occur at approximately 6-month intervals, satisfying the year-round demand for pomegranate fruit relies on postharvest storage [2]. However, pomegranate fruits are very sensitive to storage; long storage times and inappropriate conditions cause major weight and quality losses [3]. Other important problems associated with pomegranate fruit storage are chilling injury, decay, and changes in antioxidant activity.

Weight loss, chilling injury, and decay are the three main causes of quality loss in pomegranate fruits, and these three elements are differently influenced by temperature and relative humidity. Some studies have reported that the most suitable storage conditions for pomegranate fruits are temperatures of 6.5±1 °C and relative humidity of 90–95% [4–6]. When temperatures are above 5 °C, weight loss, and fruit decay increase, and when temperatures are below 5 °C, chilling injury increases. Similarly, more than 90% relative humidity increases fruit decay, while less than 90% relative humidity enhances weight loss [2]. Therefore, the storage life of pomegranate fruits are less than 3 months even in the suggested most favorable conditions.

To the best of the authors’ knowledge, most previous studies of pomegranate storage have been conducted using a storage period of 3 months, which cannot meet the year-round demand and misses the period of highest fruit prices in the global markets [2]. Fludioxonil is known to prevent mold decay [2] and have positive effects on the postharvest life of fruits, but also reported to have negative effects on human health, i.e. central nervous system [7]. On the other hand, propolis [8,9] and black seed oil [10], are reported to positively influence postharvest life in other fruits, but have not been tested in pomegranates. Therefore, the aim of the present work was to study the effects of propolis, black seed oil and fludioxonil with and without modified atmosphere packaging (MAP) on the postharvest quality of pomegranate cv. Wonderful. The studies lasted in 5 months (150 days) with monthly (30 days) measurement of quality attributes on destructive samples. For each month, quality attributes were also determined after 7 days of shelf life at 20 °C.

## Materials and methods

### Experimental details and treatments

#### Fruit samples

The fruit samples used in the present study belonged to the Wonderful cultivar, which now dominates the world pomegranate trade and consumption [2]. The fruits were harvested by hand on 26 October 2016 at commercial maturity (red color, total soluble solids approximately 17% and titratable acidity (TA) approximately 1.80%) from an 8-year-old orchard located in Güzelyurt in the northern part of Cyprus. The harvested fruits were immediately transported to Alnar Pomegranates Ltd. in a ventilated truck to protect weight loss. The fruits were categorized according to EU standards [11] and selected for uniformity in size and color. The “extra” category fruits with size “10” (for boxes of 40 cm x 30 cm dimensions) were used in the present study.

#### Propolis extract

Crude propolis was collected from a private land located in the Bağlıköy region in the western part of Cyprus. The owner of the land gave permission to collect samples from his own land and no specific permissions required for this activity. The region where the bees (*Apis mellifera cypria*) collected propolis exudates were dominated by plant species including pine (*Pinus brutia* L.), olive (*Olea europaea* L.), eucalyptus (*Eucalyptus globulus* L.), citrus (*Citrus* spp.), alfalfa and clover (*Medicago* spp. and *Trifolium* spp.), pimpernel (*Anagallis arvensis* L.), wild barley (*Hordeum bulbosum* L.), field bindweed (*Convolvulus arvensis* L.), chrysanthemum (*Chrysanthemum* spp.) and locust tree (*Acacia* spp.). Collecting of the crude propolis from the land did not damage any endangered or protected species. Preparation of the propolis extract was performed according to the method by [12] with some modifications (described below). The propolis extracts were frozen at -20 °C for 1 month, then ground in a small mill. Then, 10% ethanol extracted propolis was prepared by adding 90 mL of 70% ethanol to 10 g of propolis and agitating for 1 week. Agitation was performed automatically by shaking the extract for 1 minute at 60-minute intervals. The mixture was then filtered through Whatman 1 filter paper. The filtered solution was stored at 4 °C in darkness until use. The 0.01% and 0.1% propolis extracts were prepared by making a dilution of the final solution with pure water (instead of 70% ethanol as described by Krell [12]) in the required proportions. The preparation of the final extracts was performed with pure water to maximize practicability and usability in real applications.

#### Black seed oil

Black seed oil is a product of the *Nigella sativa* plant, which is native to Asia. The black seed oil used in the present study was marketed by Pelmur Ltd. with the brand name Biotama. Black seed oil is obtained by cold pressing black cumin seeds. The purchased black seed oil was 100% pure and was dissolved in ethanol by adding 90 mL of 70% ethanol to 10 mL of the black seed oil and agitating for 1 day. The 0.1% and 0.5% black seed oil solutions were prepared by making a dilution of the final solution with pure water as for propolis.

#### Fludioxonil

Fludioxonil (Celest Max 100 FS, 100 g a.i. L^-1^, Syngenta) is a non-systemic fungicide. The suggested application dose for fludioxonil (0.06%) was used in the present study.

#### Modified atmosphere packaging materials

The MAP bags used in the present study were obtained from Dekatrend Ltd., located in Bursa, Turkey, with the brand name Trendlife. MAP bags are manufactured from a semi-permeable film that can control gas exchange. The packaging material used was low-density polyethylene film. The packages are designed to regulate inner air composition to approximately 9–15% CO_2_ and 6–10% O_2_. The material also has the ability to vent excessive ethylene gas and relative humidity.

#### Treatments

Pomegranates were subjected to the following treatments: (1) dipping in pure water (control treatment); (2) dipping in propolis extract (0.01%); (3) dipping in propolis extract (0.1%); (4) dipping in black seed oil (0.1%); (5) dipping in black seed oil (0.5%); and (6) dipping in fludioxonil (0.06%). Each fruit was dipped for 30 s into one of the prepared solutions [13]. A packing line dryer was used to dry the treated fruits. Each treatment contained 3 replicates of 40 fruits. Half of the treated fruits were stored in MAP bags, and the other half were stored uncovered. All fruits (bagged and unbagged) were placed in corrugated cartons (40 × 30 × 12 cm) and stored at 6.5±1 °C and 90–95% relative humidity for 30, 60, 90, 120 and 150 days. The experiment was conducted according to split-plot design. The two main factors of the present study were (1) MAP (with/without) and (2) storage duration. All fruits were numbered before the experiment; fruit weights were measured and noted. Other quality measurements were also done at the first day after harvest to enable later comparison.

### Data collection

Quality measurements were made at 30-day intervals for each replication. After each storage period (30-day intervals), 2 out of 4 fruits (of each replication of unique treatments) were removed from the storage conditions and kept at 20 °C for 7 days (shelf life) and then analyzed. Fruit weight was determined by using a digital scale (sensitive to 0.01 g). Observation of gray mold was scored according to a 0–4 scale reported by Palou et al. [13]: 0, no infection; 1, mold at only the crown; 2, mold covering less than 25% of the rind; 3, mold covering 25–50% of the rind; and 4, mold covering more than 50% of the rind. Visual analysis was performed according to the 0–5 scale suggested by Silvia et al. [14], as follows: 0, dry fruits with very severe blemishes and/or widespread microorganism attack; 1, 51–60% fruit dryness and severe blemishes and/or microorganism attack; 2, 31–50% fruit dryness and/or moderate blemishes; 3, 11–30% fruit dryness and/or minor blemishes; 4, 1–10% of fruit dryness and/or blemishes; and 5, fruits with less than 1% of dents, absence of blemishes and absence of microorganism attack. Pomegranates were also scored for chilling injury according to a 0–3 scale, as follows: 0, none; 1, slight (≤25%); 2, moderate (26–50%); and 3, severe (>51%). The measurements for weight loss, visual quality, chilling injury and gray mold were made on 6 samples at each storage duration. Moreover, following chemical measuremets (antioxidant activity, anthocyanin, ascorbic acid, titratable acidity) were made on juice from 3 randomly selected samples out of 6 for each unique treatment.

#### Determination of antioxidant activity

The antioxidant activity of pomegranate juice was determined by using the 2,2-diphenyl-1-picrylhydrazyl (DPPH) free radical scavenging method. The modified method of Klimczak et al. [15] was used for the measurements. A total of 5 mL pomegranate juice was mixed with 5 mL of methyl alcohol (80%) in tubes and then centrifuged (4000 rpm, 10 min, 4 °C). Then, 0.1 mL of supernatant was added to 2.46 mL of DPPH (0.1 mg/L in 80% methyl alcohol) and mixed by vortexing. The absorbance of the samples was measured at 515 nm using the spectrophotometer after incubating the samples in the dark for 10 min. Antioxidant activity was expressed as the percentage decline of the absorbance as follows:

$$Antioxidant\;activity\;(\%)=\left(\frac{A\;control-A\;sample}{A\;control}\right)\times 100$$ (1)

where A _control_ is the absorbance of the control (without supernatant) and A _sample_ is the absorbance of the sample.

#### Determination of total anthocyanin

The total anthocyanin content of pomegranate juice was measured using the pH differential method suggested by Fuleki and Francis [16] and developed by Giusti and Wrolstad [17]. The absorbance was measured spectrophotometrically at 510 and 700 nm in buffers of pH 1.0 and 4.5. The results were expressed as mg L^-1^.

#### Determination of ascorbic acid content

The Ascorbic acid determination was performed by using high-performance liquid chromatography (HPLC) following the method of Lee and Coastes [18]. The HPLC column was maintained at 25 °C, and the flow rate was 0.5 mL min^-1^. A total of 10 μL supernatant was injected into the Xterra C18(Waters, 4.6 × 250 mm) column. The photodiode array detector was set at 244 nm, and 2% KH_2_PO_4_ (pH 2.4) was used as the mobile phase.

#### Determination of titratable acidity

The TA (g/100 g of citric acid) of the juice samples was evaluated according to [19] with a WTW pH meter (Weilheim, Germany). TA was determined by titrating 2 mL of fruit juice in 38 mL of distilled water with 0.1 N NaOH to an endpoint of pH 8.1 and the total mL of required NaOH was recorded. To convert the size of titration to the concentration of citric acid, 0.0064 was used as a multiplication factor. The full formula used for the converting is as follows:

$$TA\;(g/100\;g\;citric\;acid)=\left(\frac{(mL\;of\;NaOH\;used)\times 0.0064}{mL\;of\;sample\;used}\right)\times 100$$ (2)

### Statistical analysis

Postharvest treatments and storage durations were used as factors, and the experimental data were subjected to analysis of variance (ANOVA) with SPSS 20.0 software. Mean separation was performed by using Tukey’s (HSD) multiple range test at P≤0.05. Moreover, overall means of with and without MAP applications were compared with independent samples t-test.

## Results

### Effects on weight loss

Raw dataset of the experiments are all presented in S1 File. According to the results obtained, a significant difference exists between the weight losses of fruits stored with MAP and without MAP. Fruits stored without MAP always had higher weight losses (Table 1). The weight losses in unbagged control fruits were 4.9% at 30 days after storage (DAS) and 19.8% at 150 DAS, while those of control fruits packaged in MAP bags were 1.4% at 30 DAS and 9.1% at 150 DAS. Generally, both propolis and black seed oil were protective against weight loss. The weight loss at 150 DAS of the fruits treated with 0.5% black seed oil without MAP was determined as 10.1%, far below that of the control treatment (19.8%). On the other hand, application of 0.5% black seed oil with MAP storage provided even better control of weight loss, which measured 5.5% at 150 DAS. When fruits were removed from storage conditions and evaluated for shelf life, their weight loss increased as expected. No significant effects of fludioxonil on weight loss were observed.

**Table 1:** Effects of different treatments on weight loss (%) in pomegranate fruits during 150 days of storage.

Treatments		Weight loss (%)									
		30 DAS	30 DAS + 7 SL	60 DAS	60 DAS + 7 SL	90 DAS	90 DAS + 7 SL	120 DAS	120 DAS + 7 SL	150 DAS	150 DAS + 7 SL
**Without MAP**	**Control**	4.9 d	12.5 c	6.5 f	11.5 c	9.8 d	14.9 d	14.4 e	17.3 c	19.8 h	23.5 e
	**Propolis (0.01%)**	3.0 c	11.3 ab	5.3 e	10.5 b	7.0 c	12.9 cd	9.70 d	14.2 b	13.3 f	19.4 cd
	**Propolis (0.1%)**	2.2 bc	10.5 ab	3.8 cd	9.4 ab	5.3 b	11.8 bc	7.6 cd	13.9 b	11.3 de	17.4 c
	**Black seed oil (0.1%)**	3.1 c	12.0 ab	4.2 d	11.4 c	6.6 c	12.0 bc	8.6 d	14.0 b	11.8 ef	18.4 c
	**Black seed oil (0.5%)**	2.6 c	9.8 a	3.1 bc	9.0 ab	5.0 b	11.3 bc	7.6 cd	13.5 b	10.1 cd	16.5 bc
	**Fludioxonil (0.06%)**	4.6 d	11.7 ab	6.4 f	10.6 ab	10.0 d	14.7 d	14.0 e	17.5 c	18.0 g	21.2 de
**With MAP**	**Control**	1.4 ab	10.4 ab	2.8 b	9.7 ab	5.2 b	13.1 cd	6.1 bc	13.8 b	9.1 c	14.3 ab
	**Propolis (0.01%)**	1.1 ab	10.2 ab	1.8 a	9.1 ab	3.7 a	10.0 ab	4.8 ab	11.0 a	7.4 b	12.3 a
	**Propolis (0.1%)**	1.3 ab	9.4 a	1.6 a	8.5 a	3.4 a	9.5 a	4.4 ab	10.2 a	6.3 ab	11.7 a
	**Black seed oil (0.1%)**	1.3 ab	9.2 a	1.7 a	8.7 a	3.6 a	9.9 ab	4.6 ab	10.8 a	6.4 ab	12.1 a
	**Black seed oil (0.5%)**	1.0 a	9.1 a	1.4 a	8.2 a	3.1 a	9.3 a	4.0 a	10.1 a	5.5 a	11.6 a
	**Fludioxonil (0.06%)**	1.4 ab	11.2 ab	2.6 b	9.2 ab	5.3 b	12.9 cd	6.2 bc	13.4 b	9.3 c	13.4 a
**Without MAP (overall)**		3.4*	11.3*	4.9*	10.4*	7.3*	12.9*	10.3*	15.0*	14.1*	19.4*
**With MAP (overall)**		1.3*	9.9*	2.0*	8.9*	4.1*	10.8*	5.0*	11.6*	7.3*	12.6*

DAS: days after storage; SL: shelf life. Values followed by the same letter or letters within the same column are not significantly different at a 5% level (Tukey’s (HSD) multiple range test). Overall means for with MAP and without MAP applications was compared with independent samples t-test; significant differences was indicated with *, and ns was used to indicate non significance.

### Effects on visual quality and chilling injury

No significant difference was found between the visual quality scores at the first day of harvest and those at 30 DAS (Table 2). After that time, the visual quality scores showed considerable declines in all treatments. Similar to the other test parameters, the visual quality of the fruits was protected by MAP application. Both black seed oil and propolis treatments were also found to protect visual quality by retarding weight loss and preventing mold, drying, and chilling injury. At 150 DAS, the visual quality scores of the controls without MAP and with MAP were 0.7 and 2.5, respectively. On the other hand, the corresponding visual quality scores in the black seed oil and propolis treatments with MAP were found to be between 3.5 and 3.7. The shelf life assessment again found a considerable decline in visual quality, as in fruit weight.

**Table 2:** Visual quality (0–5 scale) and chilling injury (0–3 scale) scores in pomegranate fruits during 150 days of storage when exposed to different treatments and storage conditions.

Treatments		30 DAS	30 DAS + 7 SL	60 DAS	60 DAS + 7 SL	90 DAS	90 DAS + 7 SL	120 DAS	120 DAS + 7 SL	150 DAS	150 DAS + 7 SL
**Visual quality scores (0–5 scale)**											
***At Harvest***		***5*.*0 a***	***5*.*0 a***	***5*.*0 a***	***5*.*0 a***	***5*.*0 a***	***5*.*0 a***	***5*.*0 a***	***5*.*0 a***	***5*.*0 a***	***5*.*0 a***
**Without MAP**	**Control**	4.5 a	3.7 d	3.8 d	3.7 b	2.8 e	2.5 e	2.3 c	1.3 e	0.7 d	0.0 g
	**Propolis (0.01%)**	4.7 a	3.7 d	4.3 cd	3.8 b	3.8 d	3.7 cd	3.3 b	2.0 de	2.0 c	1.0 f
	**Propolis (0.1%)**	4.8 a	4.2 bc	4.2 cd	3.7 b	4.2 cd	3.8 bc	3.3 b	2.8 bc	2.3 c	1.2 f
	**Black seed oil (0.1%)**	4.7 a	4.0 cd	4.5 bc	3.7 b	4.3 cd	3.8 bc	3.3 b	2.2 cd	2.2 c	0.8 f
	**Black seed oil (0.5%)**	5.0 a	4.3 bc	4.8 ab	4.0 b	4.3 cd	3.7 cd	3.5 b	2.3 cd	2.3 c	1.3 ef
	**Fludioxonil (0.06%)**	4.8 a	4.3 bc	4.3 cd	3.7 b	3.7 d	3.2 d	2.3 c	1.8 de	0.8 d	0.2 g
**With MAP**	**Control**	4.8 a	4.3 bc	4.3 cd	3.8 b	4.0 cd	3.7 cd	3.5 b	3.3 b	2.5 c	1.8 de
	**Propolis (0.01%)**	4.8 a	4.3 bc	4.8 ab	4.0 b	4.8 ab	4.0 bc	3.7 b	3.2 b	3.5 b	2.2 cd
	**Propolis (0.1%)**	5.0 a	4.5 ab	5.0 a	4.2 b	4.8 ab	4.2 bc	4.0 b	3.3 b	3.5 b	2.5 bc
	**Black seed oil (0.1%)**	4.8 a	4.3 bc	4.7 bc	4.2 b	4.7 bc	4.0 bc	3.8 b	3.3 b	3.7 b	2.5 bc
	**Black seed oil (0.5%)**	5.0 a	4.7 ab	4.8 ab	4.3 b	5.0 a	4.3 b	4.0 b	3.3 b	3.5 b	2.8 b
	**Fludioxonil (0.06%)**	4.8 a	4.8 ab	4.8 ab	4.2 b	4.3 cd	4.2 bc	3.5 b	3.5 b	2.7 c	2.2 cd
**Without MAP (overall)**		4.8ns	4.0*	4.3*	3.8*	3.9*	3.4*	3.0*	2.1*	1.7*	0.8*
**With MAP (overall)**		4.9ns	4.5*	4.8*	4.1*	4.6*	4.1*	3.8*	3.3*	3.2*	2.3*
**Chilling injury scores (0–3 scale)**											
***At Harvest***		***0*.*0 a***	***0*.*0 a***	***0*.*0 a***	***0*.*0 a***	***0*.*0 a***	***0*.*0 a***	***0*.*0 a***	***0*.*0 a***	***0*.*0 a***	***0*.*0 a***
**Without MAP**	**Control**	0.0 a	0.0 a	0.3 b	0.5 b	1.3 c	1.8 e	2.3 g	2.8 f	2.7 d	3.0 f
	**Propolis (0.01%)**	0.0 a	0.0 a	0.2 ab	0.3 ab	0.7 b	1.0 d	1.8 ef	2.2 e	2.2 cd	2.2 e
	**Propolis (0.1%)**	0.0 a	0.0 a	0.0 a	0.0 a	0.5 ab	0.8 bc	1.3 cd	1.7 d	1.5 bc	2.0 de
	**Black seed oil (0.1%)**	0.0 a	0.0 a	0.0 a	0.0 a	0.3 ab	0.5 ab	1.5 de	2.2 e	1.7 bc	2.0 de
	**Black seed oil (0.5%)**	0.0 a	0.0 a	0.0 a	0.0 a	0.5 ab	0.5 ab	1.3 cd	1.7 d	1.5 bc	1.7 de
	**Fludioxonil (0.06%)**	0.0 a	0.0 a	0.3 ab	0.5 b	1.5 c	1.5 d	2.7 g	3.0 f	2.5 d	3.0 f
**With MAP**	**Control**	0.0 a	0.0 a	0.0 a	0.0 a	0.5 ab	0.8 bc	1.0 bc	1.2 bc	1.5 bc	2.2 e
	**Propolis (0.01%)**	0.0 a	0.0 a	0.0 a	0.0 a	0.2 ab	0.5 ab	0.8 bc	1.2 bc	0.7 a	1.5 cd
	**Propolis (0.1%)**	0.0 a	0.0 a	0.0 a	0.0 a	0.0 a	0.3 ab	0.5 ab	0.7 b	0.5 a	1.0 bc
	**Black seed oil (0.1%)**	0.0 a	0.0 a	0.0 a	0.0 a	0.3 ab	0.5 ab	0.7 bc	1.0 bc	0.5 a	1.0 bc
	**Black seed oil (0.5%)**	0.0 a	0.0 a	0.0 a	0.0 a	0.0 a	0.2 ab	0.5 ab	0.8 bc	0.3 a	0.7 b
	**Fludioxonil (0.06%)**	0.0 a	0.0 a	0.0 a	0.2 ab	0.5 ab	0.7 ab	1.0 bc	1.3 cd	1.3 b	1.8 de
**Without MAP (overall)**		0.0ns	0.0ns	0.1*	0.2*	0.8*	1.0*	1.8*	2.3*	2.0*	2.3*
**With MAP (overall)**		0.0ns	0.0ns	0.0*	0.0*	0.3*	0.5*	0.8*	1.0*	0.8*	1.4*

DAS: days after storage; SL: shelf life. Values followed by the same letter or letters within the same column are not significantly different at a 5% level (Tukey’s (HSD) multiple range test). Overall means for with MAP and without MAP applications was compared with independent samples t-test; significant differences was indicated with *, and ns was used to indicate non significance.

**Visual quality scores:** 5, less than 1% of dents, absence of blemishes, and absence of microorganism attack; 4, 1–10% of the fruit was blemished or dry; 3, 11–30% of the fruit was dry or showed minor blemishes; 2, 31–50% of the fruit was dry or had moderate blemishes; 1, 51–60% of the fruit was dry and had severe blemishes or microorganism attack; 0, dry with very severe blemishes or widespread microorganism attack.

**Chilling injury scores:** 0, none visible; 1, slight (≤25%); 2, moderate (26–50%); and 3, severe (>51%).

As shown in Table 2, chilling injury symptoms started to appear after 90 DAS. As expected, chilling injury symptoms increased when the fruits were moved into the shelf life phase. At 150 DAS, the chilling injury score of the control fruits without MAP was found to be 2.7 on the 0–3 scale, which indicates severe (>51%) symptoms. At the same time, the chilling injury score of the control fruits with MAP was 1.5, which means moderate (26–50%) symptoms. The best results were obtained from black seed oil and propolis applications with MAP, where the scores were measured between 0.3 and 0.7, signifying slight (≤25%) symptoms.

### Effects on gray mold development

Gray mold arising from *Botrytis cinerea* is the major cause of decay on pomegranate fruits. The most suitable conditions for gray mold are 5–10 °C and >90% relative humidity (Table 3). In both storage conditions (with and without MAP), fludioxonil treatment was found to significantly reduce the development of gray mold. However, the efficacy was of greater magnitude on fruit stored with MAP. The development of gray mold increased during storage and during the shelf life period. The gray mold score of the control fruits without MAP was determined as 3.2 at 150 DAS, while at the same time point, the gray mold score of control fruits with MAP was 1.3. Both biological and chemical treatments were found to reduce gray mold development.

**Table 3:** Gray mold decay (0–4 scale) in pomegranate fruits during 150 days of storage when exposed to different treatments and storage conditions.

Treatments		Gray mold decay (0–4 scale)									
		30 DAS	30 DAS + 7 SL	60 DAS	60 DAS + 7 SL	90 DAS	90 DAS + 7 SL	120 DAS	120 DAS + 7 SL	150 DAS	150 DAS + 7 SL
***At Harvest***		***0.0 a***	***0.0 a***	***0.0 a***	***0.0 a***	***0.0 a***	***0.0 a***	***0.0 a***	***0.0 a***	***0.0 a***	***0.0 a***
**Without MAP**	**Control**	1.5 b	1.5 d	1.8 e	2.0 f	2.2 e	2.5 f	2.3 f	3.0 e	3.2 f	3.7 f
	**Propolis (0.01%)**	0.3 a	0.7 bc	1.2 d	1.8 e	1.3 cd	1.3 e	1.7 e	2.0 cd	2.2 de	2.5 e
	**Propolis (0.1%)**	0.3 a	0.5 ab	0.8 cd	1.5 de	1.0 bc	1.3 e	1.3 de	1.5 bc	1.3 cd	1.8 d
	**Black seed oil (0.1%)**	0.3 a	0.7 bc	1.2 d	1.5 de	1.3 cd	1.3 e	1.5 e	2.2 d	1.7 de	1.8 d
	**Black seed oil (0.5%)**	0.2 a	0.3 ab	0.7 cd	1.2 cd	1.0 bc	1.2 de	1.5 e	2.2 d	1.3 cd	1.5 d
	**Fludioxonil (0.06%)**	0.0 a	0.3 ab	0.3 ab	0.3 ab	0.7 ab	1.0 cd	1.0 bc	1.2 b	0.8 bc	1.2 cd
**With MAP**	**Control**	0.3 a	0.8 c	1.0 cd	1.3 de	1.2 bc	1.3 e	1.2 cd	1.3 b	1.3 cd	1.8 d
	**Propolis (0.01%)**	0.2 a	0.5 ab	0.5 bc	0.3 ab	0.5 ab	0.8 bc	1.0 bc	1.2 b	1.2 bc	1.7 d
	**Propolis (0.1%)**	0.0 a	0.3 ab	0.3 ab	0.3 ab	0.3 ab	0.7 bc	0.5 ab	1.0 b	1.0 bc	0.8 bc
	**Black seed oil (0.1%)**	0.2 a	0.3 ab	0.5 bc	0.5 ab	0.7 ab	0.8 bc	1.0 bc	1.3 b	1.0 bc	1.2 cd
	**Black seed oil (0.5%)**	0.0 a	0.2 ab	0.3 ab	0.7 bc	0.5 ab	0.5 ab	0.7 ab	1.0 b	0.8 bc	0.7 bc
	**Fludioxonil (0.06%)**	0.0 a	0.0 a	0.0 a	0.2 ab	0.5 ab	0.3 ab	0.3 ab	0.3 a	0.5 ab	0.3 ab
**Without MAP (overall)**		0.4*	0.7*	1.0*	1.4*	1.3*	1.4*	1.6*	2.0*	1.8*	2.1*
**With MAP (overall)**		0.1*	0.4*	0.4*	0.6*	0.6*	0.8*	0.8*	1.0*	1.0*	1.1*

DAS: days after storage; SL: shelf life. Values followed by the same letter or letters within the same column are not significantly different at a 5% level (Tukey’s (HSD) multiple range test). Overall means for with MAP and without MAP applications was compared with independent samples t-test; significant differences was indicated with *, and ns was used to indicate non significance.

**Gray mold decay scores:** 0, no infection; 1, mold at only the crown; 2, mold covering less than 25% of the rind; 3, mold covering 25–50% of the rind; and 4, mold covering more than 50% of the rind.

### Effects on total antioxidant activity and anthocyanin content

The antioxidant activity of the fruits in the present study was found to be 95.23% at the first day of harvest (Table 4). The antioxidant activity of the fruit samples declined during the storage period. MAP and applications of propolis and black seed oil decreased the reduction in antioxidant activity during the first 30 days of storage. The lowest antioxidant activity at 30 DAS was found in the control and fludioxonil treatments without MAP. On the other hand, the 7-day shelf life period increased the antioxidant activity of the fruit samples after the 30^th^ day. However, the 7-day shelf life period after the 90^th^ and 150^th^ days decreased the antioxidant activities of the fruit samples.

**Table 4:** Total antioxidant activities (%) and anthocyanin contents of pomegranate fruits during 150 days of storage when exposed to different treatments and storage conditions.

Treatments		30 DAS	30 DAS + 7 SL	90 DAS	90 DAS + 7 SL	150 DAS	150 DAS + 7 SL
**Total antioxidant activity (%) “DPPH radical scavenging activity”**							
***At Harvest***		***95.2 a***	***95.2 a***	***95.2 a***	***95.2 a***	***95.2 a***	***95.2 a***
**Without MAP**	**Control**	92.5 d	94.6 b	93.5 b	93.2 b	93.1 b	92.9 b
	**Propolis (0.1%)**	93.2 c	95.1 a	93.5 b	93.3 b	93.0 b	92.8 b
	**Black seed oil (0.5%)**	93.3 c	95.3 a	93.5 b	93.3 b	93.0 b	92.9 b
	**Fludioxonil (0.06%)**	92.6 d	94.8 b	93.5 b	93.3 b	93.1 b	92.8 b
**With MAP**	**Control**	94.5 b	95.6 a	93.6 b	93.4 b	93.1 b	92.9 b
	**Propolis (0.1%)**	94.6 b	95.8 a	93.6 b	93.3 b	93.1 b	92.9 b
	**Black seed oil (0.5%)**	94.6 b	95.7 a	93.6 b	93.3 b	93.1 b	92.9 b
	**Fludioxonil (0.06%)**	94.4 b	95.5 a	93.6 b	93.3 b	93.1 b	92.9 b
**Without MAP (overall)**		92.9*	94.9*	93.5*	93.2*	93.0*	92.8*
**With MAP (overall)**		94.5*	95.7*	93.6*	93.3*	93.1*	92.9*
**Total anthocyanin content (mg/L)**							
***At Harvest***		***124*.*6 a***	***124*.*6 a***	***124*.*6 a***	***124*.*6 a***	***124*.*6 a***	***124*.*6 a***
**Without MAP**	**Control**	144.6 f	137.6 f	385.0 c	321.9 c	242.2 d	199.4 c
	**Propolis (0.1%)**	138.5 c	135.2 d	384.5 c	323.1 c	247.0 d	196.8 c
	**Black seed oil (0.5%)**	140.7 d	133.3 c	384.6 c	322.9 c	244.1 d	196.2 c
	**Fludioxonil (0.06%)**	143.5 e	137.0 e	384.1 c	323.0 c	245.7 d	194.3 c
**With MAP**	**Control**	139.2 c	134.6 d	358.7 b	293.3 b	212.0 b	171.7 b
	**Propolis (0.1%)**	133.5 b	132.4 b	357.5 b	293.0 b	215.6 c	171.7 b
	**Black seed oil (0.5%)**	133.5 b	132.0 b	356.3 b	292.1 b	215.3 c	170.4 b
	**Fludioxonil (0.06%)**	139.1 c	134.9 d	355.6 b	290.7 b	212.6 b	170.1 b
**Without MAP (overall)**		141.8*	135.8*	384.6*	322.7*	244.7*	196.7*
**With MAP (overall)**		136.3*	133.5*	357.0*	292.3*	213.8*	171.0*

DAS: days after storage; SL: shelf life. Values followed by the same letter or letters within the same column are not significantly different at a 5% level (Tukey’s (HSD) multiple range test). Overall means for with MAP and without MAP applications was compared with independent samples t-test; significant differences was indicated with *, and ns was used to indicate non significance.

The fruit samples in the present study contained a total of 124.57 mg/L anthocyanin at the first day of harvest. The total anthocyanin contents of all samples increased significantly during the first 90 days and then decreased. At the end of the experiment (day 150), the total anthocyanin contents of all samples were relatively higher than those on the first day of harvest. No significant difference was found among the control, propolis, black seed oil and fludioxonil treatments, but a significant difference was determined between the treatments with and without MAP. The fruits stored without MAP had higher total anthocyanin contents than the fruits stored with MAP during both the storage and shelf life periods.

### Effects on ascorbic acid content and titratable acidity

The initial ascorbic acid content of the pomegranate fruits was found to be 67.3 mg L^-1^ at the first day of harvest, and it significantly decreased in all treatments during storage and shelf life (Table 5). Fruit samples stored without MAP had higher ascorbic acid contents than fruits stored with MAP did. On the other hand, treatment with either propolis or black seed oil was found to prevent the reduction of ascorbic acid content. The initial TA content was determined as 1.81 g 100 g^-1^ citric acid in the present study. The citric acid content decreased in all treatments during the storage and shelf life periods.

**Table 5:** Ascorbic acid content and titratable acidity of pomegranate fruits during 150 days of storage when exposed to different treatments and storage conditions.

Treatments		30 DAS	30 DAS + 7 SL	90 DAS	90 DAS + 7 SL	150 DAS	150 DAS + 7 SL
**Ascorbic acid (mg/L)**							
***At Harvest***		***67*.*3 a***	***67*.*3 a***	***67*.*3 a***	***67*.*3 a***	***67*.*3 a***	***67*.*3 a***
**Without MAP**	**Control**	55.4 c	50.6 c	44.1 cd	35.6 c	27.4 c	18.0 c
	**Propolis (0.1%)**	57.3 b	53.2 b	48.8 b	39.8 b	32.4 b	19.1 b
	**Black seed oil (0.5%)**	57.1 b	53.3 b	48.8 b	40.5 b	31.7 b	19.1 b
	**Fludioxonil (0.06%)**	55.3 c	50.6 c	44.9 c	34.9 c	25.8 d	18.2 bc
**With MAP**	**Control**	52.3 e	46.1 e	40.1 e	30.9 e	20.8 e	12.2 d
	**Propolis (0.1%)**	54.0 d	48.3 d	43.4 d	32.5 d	20.9 e	12.9 d
	**Black seed oil (0.5%)**	53.8 d	48.1 d	43.9 d	34.3 c	21.0 e	12.8 d
	**Fludioxonil (0.06%)**	52.4 e	46.3 e	40.7 e	30.4 e	20.2 e	12.4 d
**Without MAP (overall)**		56.3*	51.9*	46.6*	37.7*	29.3*	18.6*
**With MAP (overall)**		53.1*	47.2*	42.0*	32.0*	20.7*	12.5*
**Titratable acidity (g/100 g citric acid)**							
***At Harvest***		***1*.*81 a***	***1*.*81 a***	***1*.*81 a***	***1*.*81 a***	***1*.*81 a***	***1*.*81 a***
**Without MAP**	**Control**	1.63 c	1.45 c	1.39 c	1.21 d	1.28 c	1.09 c
	**Propolis (0.1%)**	1.64 c	1.46 c	1.39 c	1.25 c	1.30 c	1.10 c
	**Black seed oil (0.5%)**	1.62 c	1.47 c	1.39 c	1.26 c	1.29 c	1.10 c
	**Fludioxonil (0.06%)**	1.63 c	1.46 c	1.40 c	1.25 c	1.31 c	1.10 c
**With MAP**	**Control**	1.73 b	1.55 b	1.60 b	1.44 b	1.52 b	1.23 b
	**Propolis (0.1%)**	1.73 b	1.57 b	1.61 b	1.46 b	1.52 b	1.24 b
	**Black seed oil (0.5%)**	1.72 b	1.55 b	1.61 b	1.47 b	1.52 b	1.23 b
	**Fludioxonil (0.06%)**	1.73 b	1.56 b	1.61 b	1.45 b	1.53 b	1.22 b
**Without MAP (overall)**		1.63*	1.46*	1.39*	1.24*	1.30*	1.10*
**With MAP (overall)**		1.73*	1.56*	1.61*	1.46*	1.52*	1.23*

DAS: days after storage; SL: shelf life. Values followed by the same letter or letters within the same column are not significantly different at a 5% level (Tukey’s (HSD) multiple range test). Overall means for with MAP and without MAP applications was compared with independent samples t-test; significant differences was indicated with *, and ns was used to indicate non significance.

## Discussion

Weight loss is an important quality attribute explaining the storage quality of fruits, and it is generally increased with storage time. Weight loss also reduces the attractiveness of pomegranate fruits by changing the shape, browning the rind and hardening the husk of the fruits [20]. Al-Mughrabi et al. [5] reported 32% weight loss in pomegranate fruits at 8 weeks when fruits were stored at 22 °C. The results of the current work are in agreement with those of previous studies, which reported a high efficacy of MAP in the prevention of weight loss [21,22]. On the other hand, fruits clearly showed higher weight loss after the shelf life period. Here, an important connection can be made to the negative effect of MAP after opening the bags. MAP establishes a microenvironment with low O_2_, high CO_2_, and high relative humidity levels, and these factors slow down respiration and transpiration. However, after the bags are opened, this microenvironment is abolished, and fruits start to lose weight rapidly. These results suggest that fruits stored with MAP should be delivered to consumers without opening the bags. The highest efficacy was found in the 0.5% black seed oil treatment when combined with MAP. The weight loss under this treatment at 150 DAS was only 5.5%. Most previous postharvest storage studies of pomegranate fruits were conducted for shorter periods than that of the present study. Furthermore, even over shorter periods, most of the previous studies [13,23] could not limit fruit weight loss to the amount achieved in the present study for longer periods of storage.

The results of the present study showed that chilling injury symptoms started to appear after 3 months of storage. This result is in accordance with the report of Elyatem and Kader [3]. Similarly to the present study, Nerya et al. [24] noted that MAP reduces chilling injury in pomegranates. As reported by Elyatem and Kader [3], the chilling injury symptoms in the present study became more noticeable when the fruits were transferred to ambient temperatures. As a result, the application of propolis or black seed oil with MAP led to a significant reduction in skin browning, with values of less than 1 (slight, ≤25%), in contrast to a value of approximately 3 (severe, >51%) in control fruits without MAP at 150 DAS. The application of propolis or black seed oil without MAP was also found to reduce chilling injury to levels below 2 (moderate, 26–50%).

Gray mold is the main cause of decay on pomegranate fruit and is mainly caused by *Botrytis cinerea* [25]. This fungus infects the flowers and/or crowns of fruits in the field, begins to develop from the crown, and then spreads to the rest of the fruit. It can also easily spread to nearby fruits during storage [20]. The most suitable conditions for gray mold development are temperatures of 5–10 °C and relative humidity higher than 90%. Tedford et al. [26] reported that without any control measures, the losses caused by gray mold could reach up to 30% in pomegranates. The development of gray mold increased during the storage and shelf life periods. These findings are in accordance with the results of Palou et al. [13], where the researchers reported that fludioxonil application reduces postharvest decay losses. Similarly, significant reduction in gray mold development has been reported by Artes et al. [27], and Nanda et al. [28] for fruits stored in MAP. In this study, a considerable effect on the development of gray mold was found in the black seed oil and propolis treatments, with or without MAP. These results indicated that both natural extracts can be used as alternatives for the control of gray mold development on pomegranate fruit. Similarly, Özdemir et al. [8] noted that ethanol-dissolved propolis reduced fungal decay on grapefruit cv. Star Ruby.

Pomegranate fruit has high phenolic contents and antioxidant properties [29]. The findings of the present study are in accordance with the report of Fawole and Opara [30], who noted 95.7% of antioxidant activity from the South African cultivar Bhagwa. The antioxidant activity of the fruit samples declined during the storage period. Similarly, Arendse et al. [31] noted an increase in the antioxidant activity of pomegranate fruits during the first months of storage and a decrease during the following months. The decline in antioxidant activity is related to enzymatic activity occurring during storage, which causes the breakdown of phenolic compounds [6]. Both MAP and applications of propolis and black seed oil prevented the reduction in antioxidant activity during the first 30 days of storage. The lowest antioxidant activities at 30 DAS were determined in the control and fludioxonil treatments without MAP. On the other hand, the 7-day shelf life period increased the antioxidant activity of the fruit samples after the 30^th^ day. However, the 7-day shelf life period after the 90^th^ and 150^th^ days decreased the antioxidant activities of the fruit samples.

Pomegranate fruit is known to be one of the major sources of anthocyanin [32]. Anthocyanins are a subclass of flavonoids that are responsible for red, purple, and blue coloration in pomegranates. Anthocyanins are highly reactive against free radicals due to their electron-deficient chemical structures, and thus they have strong antioxidant activity [33,34]. The fruit samples in the present study contained a total of 124.57 mg/L anthocyanin at the first day of harvest. The fruits stored without MAP had higher total anthocyanin contents than the fruits stored with MAP during both the storage and shelf life periods. These results are in accordance with the findings of Selcuk and Erkan [22], Artes et al. [27] and Miguel et al. [35]. Miguel et al. [35] reported that anthocyanin synthesis and/or degradation are affected by CO_2_ and O_2_ levels, and this may be the reason that fruits stored under MAP showed different responses during storage.

Fruit storage duration is believed to cause a decline in the concentration of ascorbic acid [36]. In the present study, the fruit samples stored without MAP had higher ascorbic acid contents than the fruits stored with MAP did. On the other hand, applications of both propolis and black seed oil were found to prevent the reduction of ascorbic acid content. The irreversible oxidation of dehydro-L-ascorbic acid (DHAA) during storage might be the reason for the decrease in ascorbic acid content. Moreover, the reduction of the ascorbic acid content under MAP and propolis or black seed oil treatment might be due to the fast degradation of ascorbic acid [37]. The results of the present study were in agreement with the findings of Selcuk and Erkan [22], Kulkarni and Aradhya [38] and Zarei et al. [39].

The TA of pomegranate juice is reported to differ among growing regions, maturity levels and cultivars [30]. The TA of the samples in the present study decreased in all treatments during the storage and shelf life periods. Similarly, Artés et al. [34] reported that 7 days of shelf life after cold storage causes a decrease in TA. The fruit samples stored under MAP conditions had higher TAs than those stored without MAP did. These results are in accordance with the findings of Selcuk and Erkan [22]. None of the tested chemicals caused a significant effect on TA. Similarly, El-Badawy et al. [40] reported that propolis treatment protected the weight of navel oranges but had no effect on the total acidity at doses below 2%.

## Conclusions

Both black seed oil and propolis treatments significantly affected the maintenance of fruit weight, juice content and visual quality. The effects of both treatments were increased when they were combined with MAP. Application of either 0.5% black seed oil or 0.1% propolis combined with MAP was found to protect the quality and marketability of pomegranate fruits for 150 days at 6.5±1 °C and 90–95% relative humidity. The present study also showed that applications of both black seed oil and propolis, especially when combined with MAP, were effective in controlling gray mold development (as an alternative to fungicide application) and slowed the occurrence of chilling injury, even at 150 DAS. Furthermore, shelf life significantly reduces the marketability of pomegranates, even those stored for a shorter period with or without MAP, and conveying the fruits to the final consumers in MAP bags is an important issue for the protection of fruit quality.

## Supporting information

S1 File Raw dataset for fruit weight, visual quality scores, chilling injury, gray mold decay, antioxidant activity, anthocyanin content, ascorbic acid and titratable acidity. (XLSX)
